# Prevalence of *bla*_CTX-M_ and *bla*_TEM_ Genes in Cefotaxime-Resistant *Escherichia coli* Recovered from Tertiary Care at Central Nepal: A Descriptive Cross-Sectional Study

**DOI:** 10.1155/2024/5517662

**Published:** 2024-01-08

**Authors:** Rani Kumari Sah, Pragyan Dahal, Ranjana Parajuli, Gorkha Raj Giri, Era Tuladhar

**Affiliations:** ^1^National College, Kathmandu, Nepal; ^2^Grande International Hospital, Kathmandu, Nepal

## Abstract

Urinary tract infections (UTIs) are highly prevalent globally, and various antibiotics are employed for their treatment. However, the emergence of drug-resistant uropathogens towards these antibiotics causes a high rate of morbidity and mortality. This study was conducted at the Microbiology Laboratory of Grande International Hospital from November 2021 to May 2022 and aimed to assess the prevalence of UTI caused by *Escherichia coli* and their antibiotic susceptibility pattern with a focus on extended-spectrum beta-lactamases (ESBLs) and the prevalence of two genes (*bla*_CTX-M_ and *bla*_TEM_) in cephalosporin-resistant *E. coli*. Altogether, 1050 urine samples were processed to obtain 165 isolates of *E. coli*. The isolates were identified by colony morphology and biochemical characteristics. Antimicrobial susceptibility tests (ASTs) were determined by the Kirby–Bauer disk diffusion method, and their ESBL enzymes were estimated by the combined disk method (CDM). Two ESBL genes (*bla*_CTX-M_ and *bla*_TEM_) were investigated by polymerase chain reaction (PCR) in cefotaxime-resistant *E. coli*. Among the 1050 urine samples that were processed, 335 (31.9%) were culture-positive with 165 (49.2%) identified as *E. coli.* The age group ≥60 years (30.3%) had greater susceptibility to bacterial infections. AST revealed that meropenem was highly effective (95.7% susceptibility), while ampicillin showed the least sensitivity (42.4%). Among the *E. coli* isolates, 86 were multidrug resistant (MDR) and 10 were extensively drug resistant (XDR). Of these, 46 MDR (96%) and 2 XDR (4%) were ESBL producers. The prevalence of ESBL genes (*bla*_CTX-M_ and *bla*_TEM_) was 49.3% and 54.8%, respectively. The overall accuracy of CDM as compared to PCR for the detection of the *bla*_CTX-M_ gene was 55.26%. The prevalence of MDR *E. coli* harboring the *bla*_CTX-M_ and *bla*_TEM_ genes underscores the imperative role of ESBL testing in accurately identifying both beta-lactamase producers and nonproducers.

## 1. Introduction

The presence of bacteria in the urine can range from asymptomatic to a serious kidney infection with sepsis [1]. In community medicine, urinary tract infections (UTIs) are the second most frequent infection [2]. UTI has emerged as a significant and urgent public health issue. An estimated 150 million cases of UTI, which have a significant risk of morbidity and mortality, are detected each year [3]. Males and females of all ages are both affected by UTIs. Females are more prone to experience UTIs than males, which is probably due to anatomical variations, hormonal influences, and behavioral factors [4].

UTI cases range from 23.1% to 37.4% among Nepalese patients visiting general hospitals. More than 95% of UTI instances are due to bacteria, which is a prevalent cause. *Escherichia coli* causes more than 80% of UTIs and is the most frequent bacteria [5]. It is well-recognized that UTIs can result in kidney scarring permanently as well as short-term morbidities such as fever, dysuria, and lower abdominal pain (LAP) [6]. Altogether, 50% of all nosocomial infections and 70% to 95% of uropathogenic *E*. *coli* (UPEC) are considered the source of community-acquired UTIs [7].

*E. coli* predominates in a higher extent of UTI cases, followed by *Proteus* species*, Staphylococcus saprophyticus, Klebsiella* species, and other *Enterobacteriaceae* families [8]. A significant public health concern is the development of antibiotic resistance in the treatment of UTIs. Counterfeit and spurious pharmaceuticals of uncertain quality are widely available, notably in underdeveloped nations where hunger, illiteracy, and poor hygiene habits are highly prevalent [9].

The most researched class A beta-lactam enzymes in Gram-negative bacteria are the TEM and CTX-M beta-lactamases, which are mainly plasmid-borne [10]. They are thought to be the most typical beta-lactam-resistant mechanism in Gram-negative bacilli and are spreading quickly throughout the world [11]. Based on the antibiotic resistance profile of the urinary pathogens from the most recent surveillance data, treatment for UTI cases is frequently initiated empirically. Thus, the purpose of the current investigation was to ascertain the prevalence of the *bla*_CTX-M_ and *bla*_TEM_ genes in cefotaxime-resistant *E. coli* isolated from urine samples of UTI-diagnosed patients who had visited Grande International Hospital in Kathmandu, Nepal.

## 2. Methodology

### 2.1. Study Design, Site, and Criteria

This hospital-based descriptive cross-sectional study was carried out at Grande International Hospital, and molecular assays were conducted at National College, Kathmandu, at the Department of Microbiology, Kathmandu, Nepal, on the duration of November 2021 to May 2022.

#### 2.1.1. Inclusion Criteria

Samples from patients of all age groups for evaluation of urinary tract infections referred by the clinician were accepted, and the isolated *Escherichia coli* isolates were included in our study.

#### 2.1.2. Exclusion Criteria

Isolates other than *Escherichia coli* were excluded.

### 2.2. Sample Size and Sampling Technique

For routine culture and antibiotic susceptibility testing, 1050 urine samples referred by clinicians were processed, and altogether, 165 *Escherichia coli* isolates were selected using a convenience sampling method.

### 2.3. Sample Collection and Transportation

Patients were instructed to fill a sterile, dry, wide-necked, leak-proof container with 10–20 ml of the clean-catch midstream region of their urine. The container was correctly labelled with the sample code, name, date, and collection time, and it was immediately transported to the microbiology laboratory. For delayed processing, the urine samples were subjected to 10% boric acid [12].

### 2.4. Laboratory Processing of the Specimen

#### 2.4.1. Urine Culture

The urine samples were inoculated on the surface of cystine lactose electrolyte deficient (CLED) agar plates, using a standard calibrated loop (∼4 mm diameter). The semiquantitative bacteriuria count was determined to estimate a significant UTI. On the surface of the culture medium, a loop of urine was streaked, and it was then incubated for 24 hours at 35°C under aerobic conditions. The total count of colonies was used to calculate the colony-forming unit (CFU) per milliliter of urine. The bacterial count was reported as significant for >10^5^ CFU/ml. If the specimen was found to be contaminated, then a repeat sample was requested [13].

### 2.5. Identification of the Isolates

According to Bergey's Manual of Systemic Bacteriology, standard microbiological methods such as the study of colony morphology, Gram's staining, and other biochemical tests (catalase test, oxidase test, Triple Sugar Iron (TSI) test, Sulphide Indole Motility test (SIM), Urease test, Citrate utilization test, Methyl Red test, Voges–Proskauer test, Sorbitol tests, and so on) were used to identify *E. coli* [14].

### 2.6. Antibiotic Susceptibility Test

Isolated organisms were preceded by an antibiotic susceptibility test (AST) following CLSI guidelines recommendation. The antibiotics used were ampicillin (10 *μ*g), amikacin (30 *μ*g), cotrimoxazole (25 *μ*g), ciprofloxacin (5 *μ*g), nitrofurantoin (300 *μ*g), norfloxacin (10 *μ*g), ceftazidime (30 *μ*g), gentamicin (30 *μ*g), cefotaxime (30 *μ*g), and meropenem (10 *μ*g). The Kirby–Bauer disk diffusion method was used to conduct the *in-vitro* susceptibility test. In this approach, the test organism's broth culture was uniformly spread out across the surface of Mueller Hinton agar (equivalent to McFarland (0.5; inoculum density: 1.5 × 10^8^ organisms/ml)). The medium was covered with the appropriate antibiotic disks, which were then incubated for 18 hours at 37°C. Following incubation, the inhibition zone was measured using a measuring scale in mm, and the zones were compared using established interpretive criteria based on CLSI guidelines recommendations to determine whether they were susceptible, intermediate, or resistant. *E. coli* ATCC 25922 was utilized to standardize the drug susceptibility test and to check antibiotic disk quality control. [15].

### 2.7. Screening of Multidrug Resistant (MDR), Extensive Drug Resistance (XDR), and Pandrug Resistance (PDR)

In the study, isolates were classified as MDR if they were resistant to at least three classes of antibacterial agents, whereas the isolates resistant to at least one agent of all antimicrobials but susceptible to only one or two antibacterial groups were classified as XDR. Finally, isolates that were still resistant to all commercially available antibacterial agents were classified as PDR [16].

### 2.8. Phenotypic Detection of ESBL Production

The antibiotic cefotaxime (CTX, 30 *μ*g) disks was used to conduct the initial screening test for the ESBL producer. According to CLSI 2017, isolates were further tested for ESBL production only if the zone of inhibition of cefotaxime had a diameter of less than 25 mm [13]. A combination disk test utilizing cefotaxime (30 *μ*g) and cefotaxime/clavulanic acid disks (30/10 *μ*g) was performed on the *E. coli* that were not susceptible to cefotaxime. The zones of inhibition for the cefotaxime and cefotaxime/clavulanic acid disks were measured. Isolates were defined as ESBL producers when there was an increase in zone diameter of 5 mm when clavulanic acid was present compared to individual disks [16].

### 2.9. Conservation of the Isolates

For further molecular analysis, the *E. coli* isolates were stored in tryptic soy broth with 20% glycerol at −70°C.

### 2.10. DNA Extraction of *E. coli* Isolates

DNA extraction of bacterial isolates was carried out from cefotaxime (CTX)-resistant *E. coli* using boiling lysis. For this, the preserved bacteria were subcultured on nutrient agar (NA) and were incubated for 24 hrs at 37°C. A Luria Bertani (LB) broth was used to inoculate the isolated colony from the NA, and it was then incubated at 37°C. The DNA was extracted and then suspended in 50 *μ*L of TE buffer, which was later maintained at deep freeze (−20°C) for preservation [17, 18].

### 2.11. DNA Amplification and Detection

To identify the presence of ESBL genes, conventional PCR was employed for PCR amplification. A master mix including 200 *μ*M of dNTPs (dATP, dCTP, dGTP, and dTTP), 120 nM of each primer (forward and reverse), 2.5 U of Taq polymerase in 1 × PCR buffer, 25 mM of MgCl_2_, and 3 *μ*L of DNA template was added to a 21 *μ*L volume to perform PCR amplification operations. The following temperature and cycling settings were used: for *bla*_TEM_ gene (F.P: 5′-GAGACAATAACCCTGGTAAAT-3′R.P:5′-AGAAGTAAGTTGGCAGCAGTG-3′) and for *bla*_CTX-M_ gene (FP: 5′-GAAGGTCATCAAGAAGGTGCG-3′, RP: 5′-GCATTGCCACGCTTTTCATAG-3′) [19]. For both *bla*_TEM_ and *bla*_CTX-M_ genes, amplification conditions were initial denaturation at 94°C for 5 min, 35 cycles of 95°C for 1 minute, 56°C for 45 sec, 72°C for 1 minute, and a final extension at 72°C for 7 min [20, 21]. To detect amplified genes, 10 *μ*L of each reaction were subjected to gel electrophoresis by 2% agarose gel containing ethidium bromide (5 *μ*g/mL) for 1 h at 100 V in 0.5 × TBE buffer. Then, a UV transilluminator was used to see the amplified DNA bands. *bla*_TEM_ amplicon size was 459 bp, whereas as *bla*_CTX-M_ amplicon size was 560 bp [20–22]. The known positive bacterial strains for CTX-M and TEM genes were run separately as a positive control for the PCR amplification process, and the sterile water was used as a negative control'.

### 2.12. Diagnostic Comparison of Phenotypical Method with Molecular Method

The following formula was used to compute the sensitivity (SE), specificity (SP), positive predictive value (PPV), negative predictive value (NPV), and accuracy (Ac) to proceed with the evaluation of phenotypical detection when compared with PCR methods:

(1) $$\begin{array}{c} \text{Sensitivity}\left(S_{E}\right)=\frac{\text{TP}}{\left(\text{TP}+\text{FN}\right)}\times 100\%,\\ \text{Specificity}\left(S_{P}\right)=\frac{\text{TN}}{\left(\text{TN}+\text{FP}\right)}\times 100\%,\\ \text{Positive Predictive Value}\left(\text{PPV}\right)=\frac{\text{TP}}{\left(\text{TP}+\text{FP}\right)}\times 100\%,\\ \text{Negative Predictive Value}\left(\text{NPV}\right)=\frac{\text{TN}}{\left(\text{TN}+\text{FN}\right)}\times 100\%,\\ \text{Accuracy}\left(\text{Ac}\right)=\frac{\left(\text{TP}+\text{TN}\right)}{\left(\text{TP}+\text{TN}+\text{FP}+\text{FN}\right)}\times 100\%, \end{array}$$

where TP is the True Positive, TN is the True Negative, FP is the False Positive, and FN is the False Negative [23].

### 2.13. Data Processing and Statistical Analysis

All the raw data of experiments was entered in an MS Excel sheet. The data analysis was carried out using SPSS version 20 (Statistical Package for Social Science). All the numerical data were presented as simple descriptive data. The comparison of antimicrobial-resistant data with beta-lactamases and non-beta-lactamase producers as well as the comparison of the drug-resistant pattern (MDR and XDR) with ESBL producers and nonproducers was performed using the chi-square or Fisher exact test. To determine the significance of the outcome, a *p* value less than or equal to 0.05 was considered statistically significant.

## 3. Results

### 3.1. Distribution of Bacteria in a Urine Sample

Among 1050 urine specimens processed, the number of samples containing bacterial growth in culture media was found to be 335/1050 (31.9%). Among 335 bacterial growth, there were 165/335 (49.2%) growth of *Escherichia coli*, whereas 170/335 (50.7%) growth was observed for bacteria other than *E. coli* and the remaining 715 (68%) out of a total of 1050 samples were found to be sterile.

### 3.2. Age-Wise and Gender-Wise Distribution of Cases

**Figure 1:**
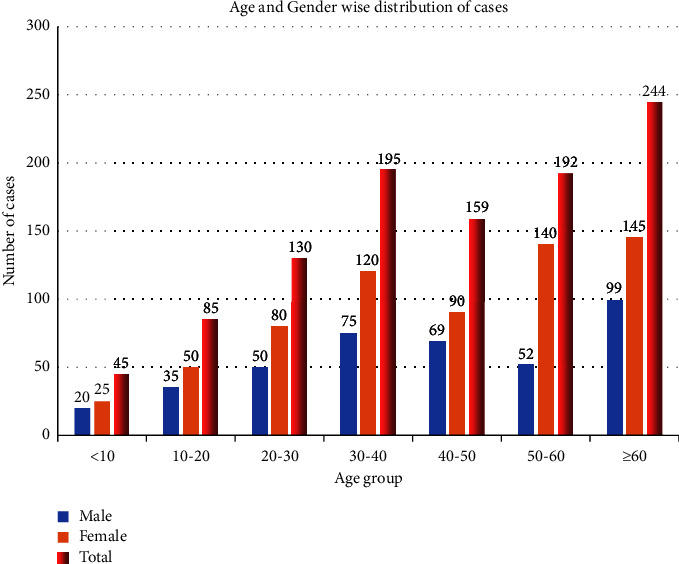
Age and gender-wise distribution of cases.

Out of 1050 samples, 400 (38%) received from male patients, while 650 (61.9%) were from female patients. The female participants of all age groups showed the highest number of participants belonging to the age group of ≥60 years was 240 (22.9%), whereas the lowest number of participants falling under <10 years was 4 (2.3%) (Figure 1).

### 3.3. Antibiotic Susceptibility Pattern of *E. coli* Isolates

Antimicrobial susceptibility tests of *Escherichia coli* obtained from urine were carried out using the Kirby–Bauer disk diffusion method. Among *E. coli* isolates, 95.7% were sensitive to meropenem, followed by gentamicin (86.7%), and nitrofurantoin (84.8%). Similarly, 64.2% and 55.7% of the isolates were sensitive to amikacin and cefotaxime, respectively. However, 57.6% and 53.4% of *E. coli* isolates showed resistance against ampicillin and cotrimoxazole. The sensitive and resistant rates are elucidated in Table 1.

### 3.4. Antibiotic Resistance Pattern of ESBL-Producing and Nonproducing *E. coli* Isolates

The antibiotics resistance pattern that was obtained by disk diffusion tests was compared with the ESBL detected by the combined disk diffusion method. The percentage of *Escherichia coli* isolates that produced ESBLs by the combined disk diffusion method was around 30.3% (50/165) while the percentage of isolates that did not produce ESBLs was determined to be 69.7% (115/165). The resistant pattern of each applied antimicrobial disk was compared with ESBL producer and non-ESBL producer *Escherichia coli* to estimate the significant difference in resistant rates for each antibiotic. According to our results, a significant difference in resistant rates was observed in the cases of ampicillin, cefotaxime, gentamicin, and meropenem which were found to be statistically significant (*P* < 0.05). The results are summarized in Table 2:

### 3.5. Multidrug Resistance (MDR) and Extensive Drug Resistance (XDR) among *E. coli* Isolates

Out of 165 *E. coli* isolates, 86 (52.1%) were found to be multidrug resistant (MDR), while 10 (6%) were extensive drug resistant.

### 3.6. ESBL Producer and Nonproducer among *E. coli* Isolates

Among 165 (*E. coli*) isolates, 73 were suspected ESBL producers on primary screening by using a cefotaxime disk; altogether, 50 were confirmed as ESBL producers by phenotypical confirmation using the combined disk diffusion method. The predominant ESBL producer was observed in *E. coli*, representing 68.5% of total cases.

### 3.7. Statistical Relationship of ESBL with MDR and XDR

Among 165 *E. coli* isolates, 50 (30.3%) were found as ESBL producers, of which 96% were MDR and 4% were XDR. Throughout the proportion, significant associations (*P*=0.001 <0.05) were observed in MDR and XDR patterns when compared with the ESBL producers *Escherichia coli*. The results are depicted in Table 3.

### 3.8. Molecular Prevalence of *bla*_CTX-M_ and *bla*_TEM_ Gene among Cefotaxime-Resistant *E. coli* Isolates

Among 50 ESBL-producing *E. coli* isolates, 50%, 46%, and 26% harbored *bla*_CTX-M_, *bla*_TEM_, and *bla*_CTX-M_ + *bla*_TEM_ genes, respectively. However, among the 23 non-ESBL-producing *E. coli* isolates (cefotaxime resistant), 47.8%, 73.9%, and 47.8% showed positive amplification of *bla*_CTX-M_, *bla*_TEM_, and *bla*_CTX-M_ + *bla*_TEM_ genes, respectively (Table 4).

**Figure 2:**
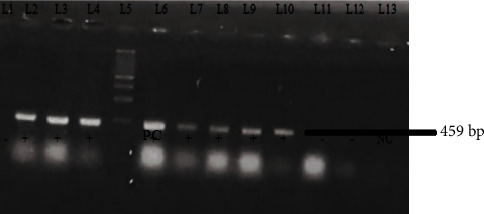
Gel electrophoresis of bacterial DNA amplification of *bla*_TEM_ gene. Marker: lane 5, 500 bp DNA ladder. Negative control: lane 13 (sterile water). Positive control: lane 6. Positive: lane 2, 3, 4, 7, 8, 9, and 10 for *bla*_TEM_ gene. Negative: lane 1, 11, and 12 for *bla*_TEM_ gene.

**Figure 3:**
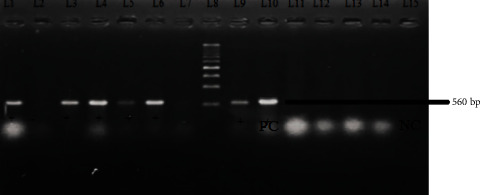
Gel electrophoresis of bacterial DNA amplification of *bla*_CTX-M_ gene. Marker: lane 8, 500 bp DNA ladder. Negative control: lane 15 (sterile water). Positive control: lane 10. Positive: lane 1, 3, 4, 5, 6, and 9 for *bla*_CTX-M_ gene. Negative: lane 2, 7, 11, 12, 13, and 14 for *bla*_CTX-M_ gene.

**Figure 4:**
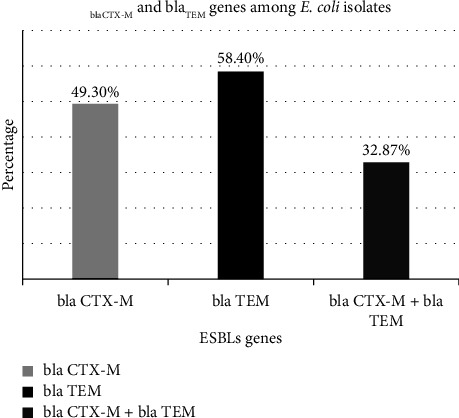
Prevalence of *bla*_CTX-M_ and *bla*_TEM_ genes among *E. coli* isolates.

To determine the prevalence of the *bla*_CTX-M_ and *bla*_TEM_ genes, bacterial DNA amplification was carried out using the conventional PCR method. The amplification of *bla*_CTX-M_ and bla_TEM_ genes is shown in Figures 2 and 3. Among 73 cefotaxime-resistant *E. coli* isolates, the *bla*_CTX-M_, *bla*_TEM_, and both (*bla*_CTX-M_ + *bla*_TEM_) genes were found to be 49.3%, 54.8%, and 32.87%, respectively. The prevalence of *bla*_TEM_ gene was subsequently greater than that of bla_CTX-M_ genes and both co-producer genes (*bla*_CTX-M_ + *bla*_TEM_). The order of magnitude based on the comparison in the prevalence of these genes is represented as *bla*_TEM_ > *bla*_CTX-M_ > *bla*_CTX-M_ + *bla*_TEM_ which is depicted in Figure 4.

### 3.9. Correlation of Phenotypical and Molecular Method concerning *bla*_CTX-M_ Genes of *E. coli*

Altogether, *n* = 73 cephalosporin-resistant *E. coli* isolates were subjected to conventional PCR and the comparison was made with the phenotypical method in the context of *bla*_CTX-M_ gene-associated ESBL. A total of *n* = 73 cefotaxime-resistant *Escherichia coli* resemble the presence of *bla*_CTX-M_ and *bla*_TEM_ genes. Twenty-five isolates of *E. coli* were phenotypically detected by combined disk diffusion method from 35 *E. coli* isolates that were positive for *bla*_CTX-M_ gene by PCR. The sensitivity and specificity of a phenotypic method when compared with PCR in terms of bla_CTX-M_ genes were 69.44%, 95% CI (51.89% to 83.65%), and 42.50%, 95% CI (27.04% to 59.11%), respectively, whereas positive predictive value (PPV) and negative predictive value (NPV) were found to be 52.08% and 60.71%. Hence, the overall accuracy of the phenotypic method for ESBL concerning *bla*_CTX-M_ gene was found to be 55.26%, with a 95% CI (43.41% to 66.69%). The results are summarized in Table 5.

## 4. Discussion

Urinary tract infection (UTI) severity varies, ranging from asymptomatic bacteria ascending to the kidneys, which can subsequently lead to sepsis [24]. In a community, UTIs are the second most common infection; elder people are more prone to it because of less immunity, decreased secretions of different hormones, poor sanitation, and so on [25].

In our study, 165 (15.7%) urine samples isolated *E*. *coli* in culture. *E. coli* were the highest prevalent bacteria from the urine sample which was well represented by a study conducted in Nepal by Poudel et al., in which the prevalence of *E. coli* was estimated to be 42.4% [26]. Many other researchers conducted in Nepal have reported a higher prevalence of *E. coli* [19, 21, 22, 24]. A comparable study conducted by Batra et al. showed 23.6% culture growth among various specimens [27]. However, in our study, the growth was found to be inferior to the study carried out by Kateregga et al., where the growth was 46.9%. Administration of previous antimicrobial therapy may hinder the ability of the organism to flourish in culture media [28].

During antibiotic susceptibility testing, antibiotics of different classes were tested against the isolates. Among 165 *E. coli* isolates, 95.7% and 86.7% were sensitive toward meropenem and gentamicin, respectively. This finding synchronizes with the findings of Sah et al. and Parajuli et al., who reported that their susceptibility towards meropenem was more than 80% [29, 30]. Another study reported that 86.4% of *E. coli* was found to be sensitive to carbapenem drugs such as meropenem, followed by gentamicin (72.8%), and amikacin (66%) which was nearly similar to our study because carbapenems are utilized as a supplementary therapy option for infections brought on by MDR Gram-negative pathogens [31, 32]. Our study, compared to other research that differs in sample types, sample sizes, organism growth rates, and antibiotic resistance patterns, may account for the proportion of sensitive isolates in both investigations.

In our study, out of 165 *E. coli* isolates, 85 (51.5%) showed multidrug resistant (MDR), and 10 (6%) showed extensive drug resistance. The present finding of MDR was nearly similar to the previous finding done at Bir Hospital in Nepal, where the rate of MDR was equivalent to 67.4% [33]. However, the present findings of MDR were comparatively lower than the findings compared to studies [30–34]. Patients who misuse, overuse, and inappropriately use antimicrobial therapy raise the cases of MDR. The primary contributing factor to the greater multidrug-resistant pattern may be incorrect antibiotic treatments from general practitioners, nurses, or over-the-counter medications, typically given in inappropriate doses, before presenting to the hospital [34]. The antibiotic-resistant patterns of *Escherichia coli* have been significantly associated with the presence of various beta-lactamase genes and the resistance traits for quinolones and aminoglycosides in the plasmid. Antibiotic resistance is primarily caused by switching the target sites, drug inactivation, modification enzymes, and the pump efflux system [35, 36].

In our study among *E. coli* isolates, 30.3% were ESBL producers which are similar to the findings by Shilpakar et al., who reported 35.5% of ESBL [37]. Another study by Uc-Cachon et al. [38] reported 83.13% ESBL among *E. coli* isolates which was higher than our study. Similarly, in the report by Poudyal et al., the ESBL-producing *E. coli* was as high as 80% [39]. Another study in Nepal showed 34.5% ESBL, where 33.3% were observed in *E. coli* which was a similar finding with our study [40]. According to a study by Shristi et al., the prevalence of *E. coli* that produces ESBLs was just 18.2% [41]. The several patients enrolled in the research, including outpatients, inpatients, patients in intensive care units (ICUs), and patients with various underlined diseases, could be one explanation for the apparent variation in different research studies that have been conducted internationally. These characteristics of hospitalized patients allow for significant factors that contribute to antibacterial resistance. These elements may involve intrusive methods and technologies, the administration of broad-spectrum antibiotics regularly, more patients who frequently have co-morbid conditions, and extended hospital stays [42].

Our study revealed the major common ESBL genes were TEM and CTX-M in *E coli* isolates that were resistant to cefotaxime, and *bla*_TEM_ (54.8%), was in strong concordance with an Indian study [43]. The most prevalent ESBL gene among *Enterobacteriaceae* has also been identified as the *bla*_CTX-M_ gene [43–45]. Multiple gene occurrences within the same organism were also seen, with *bla*_TEM_ + *bla*_CTX-M_ (32.87%) being the most prevalent [19]. These genes are typically found on plasmids or chromosomal DNA [43, 44]. The presence of the gene in plasmids further facilitated its transfer to different species of bacteria. The TEM beta-lactamase was the first plasmid-mediated enzyme, from which many of the ESBL have been derived as TEM can hydrolyze the third generation of cephalosporins, particularly ceftazidime. At the beginning of the 21st century, a new class of ESBLs on plasmids dubbed *bla*_CTX-M_ that preferentially hydrolyze cefotaxime became predominant in European countries and started to spread in Southeast Asia [44]. Monitoring of ESBLs should not be limited to phenotypic screening since there have been reports illustrating the disparity between phenotypic and genotypic detection [45]. Our study is focused on the isolation of plasmids from ESBL-producing and non-ESBL-producing *E. coli* isolates and PCR detection of plasmid-borne beta-lactamase genes (*bla*_TEM_ and *bla*_CTX-M_) that confer antibiotic resistance phenotype. The relative distribution of each gene among the *E. coli* isolates studied was analyzed and the presence of ESBL-associated genes which phenotypically non-ESBL isolates was evaluated. The presence of ESBL genes (CTX-M) in phenotypically undetected cases among tested *E. coli* isolates may confer ESBL phenotype to pathogens after acquiring the needed mutation. It is therefore necessary to consider prevalent ESBL genes (*bla*_CTX-M_) in phenotypically undetected cases making appropriate steps to address problems associated with the increasing frequency and rapid spread and horizontal gene transfer of ESBL-producing bacteria. Consequently, the new information can be useful in developing accurate protocols for detecting ESBLs and treating infections. While comparing the efficiency of phenotypical test keeping PCR detection as a reference method, our study revealed a sensitivity of 69.4% and a specificity of 42.5%, with an overall accuracy of 55.2%. The proportion of sensitivity differs based on study, sample size, burden of ESBL, and nature of genes that are recognized by phenotypical methods. In our study, the specificity might have been lowered compared to sensitivity, and we assume it is because of the presence of ESBL genes in phenotypical undetected strains of *Escherichia coli* that were resistant to cefotaxime.

This study revealed data on the growing dominance of MDR-*E. coli* and ESBL in Nepal. A previous study conducted by Regmi et al. in Nepal also revealed that 63.04% of MDR was found in *E. coli* [46]. Another study conducted by Manandhar et al. estimated 62% of MDR in *E. coli* and 16 ESBL-producing *E. coli* out of 19 ESBL-producing Gram-negative bacteria in Kathmandu, Nepal [47] that determined increasing trends of MDR and ESBL producing in *Escherichia coli*. According to our study, 50.6%, 54.8%, and 32.8% of the 73 cefotaxime-resistant *E. coli* isolates had the genes *bla*_CTX-M,_*bla*_TEM_, and *bla*_CTX-M_ + *bla*_TEM_, suggesting that these genes could transfer from one healthcare to other hospitals while shifting the patients. It aids in the ongoing monitoring and surveillance of both the genes coding for antibiotic resistance and other traits for it for better treatment options, stops the use of unnecessary antibiotics to stop the spread of antibiotic-resistant *E. coli*, and increases the number of antibiotics available for their effective use in protecting future generations [48]. The primary causes of prolonged infection, increased hospitalization, a higher cost of medication, and increased morbidity and mortality rates are often identified as bacteria that are becoming increasingly resistant to regularly used antibiotics [49]. Antibiotic usage guidelines at the national level should be developed and implemented. The use of antibiotics should be addressed only when required by medical professionals.

## 5. Strengths and Limitations

The prevalence of *E. coli*, their antibiogram, and the status of MDR, XDR, and PDR in urine samples from our study in a single hospital setting can be useful additional reference data for the existing literature and treating physicians. This study's examination of the incidence of resistant genes in ESBL-producing *E. coli* emphasizes the value and necessity of molecular diagnostic facilities for a more accurate identification of infectious illnesses. However, there are some limitations in our study as well. The prevalence of AMR cannot be generalized, as our study's methodology was exclusive to a single institution and used small clinical samples. We were unable to characterize the resistance genotypes of other Gram-negative organisms because of the limited laboratory resources and financing. Furthermore, our study did not encompass the characterization of genotypes for other Ambler class beta-lactamases, and we did not examine the presence of other ESBL gene members, including those within the SHV family.

## 6. Conclusions

*E. coli* was the most predominant in our current investigation. Females were highly infected as compared to males. Meropenem and gentamicin were found to be more sensitive towards *E. coli* isolates, while ampicillin was least effective. The multidrug-resistant and extensively drug-resistant *E. coli* increases complications in treating UTIs. The application of the PCR method in our study determined that even non-ESBL producers by the phenotypical method harbor ESBL genes such as TEM, CTX-M, and both co-producers that was very crucial information which concluded that the molecular methods also should be performed simultaneously with phenotypical methods on cefotaxime-resistant strains. Nevertheless, the phenotypical methods were easy to perform, cheap, and have good sensitivity for detection of ESBL and are still recommended by the CLSI Guidelines which could be useful for detection in the least developed countries like ours. To accurately estimate the burden of AMR, it is advised that future research involving a larger sample size and more detection of a larger number of different genes can sufficiently improve detection of missed cases of ESBL by the phenotypic method.

## Figures and Tables

**Table 1:** Antibiotic susceptibility pattern of *E. coli* isolates (*n* = 165).

Antibiotics	Sensitive		Resistant	
	*N*	%	*N*	%
Ampicillin (10 *μ*g)	70	42.4	95	57.6
Norfloxacin (10 *μ*g)	82	49.7	83	50.3
Nitrofurantoin (300 *μ*g)	140	84.8	25	15.2
Cotrimoxazole (25 *μ*g)	77	46.6	88	53.4
Cefotaxime (30 *μ*g)	92	55.7	73	44.2
Amikacin (30 *μ*g)	106	64.2	59	35.8
Meropenem (10 *μ*g)	158	95.7	7	4.2
Gentamicin (10 *μ*g)	143	86.7	22	13.3

**Table 2:** Comparison of antibiotic resistance pattern with phenotypically detected ESBL cases in *E. coli* isolates.

Antibiotics	ESBL producer		ESBL nonproducer		*P* value
	*N*	%	*N*	%	
Ampicillin (10 *μ*g)	39	78	56	48.7	<0.001
Norfloxacin (10 *μ*g)	31	62	52	45.2	0.048
Nitrofurantoin (300 *μ*g)	5	10	20	17.4	0.224
Cotrimoxazole (25 *μ*g)	27	54	60	52.1	0.829
Cefotaxime (30 *μ*g)	50	100	23	20	<0.001
Amikacin (30 *μ*g)	38	76	21	18.3	<0.001
Meropenem (10 *μ*g)	0	0	7	6	0.075
Gentamicin (10 *μ*g)	11	22	11	9.6	0.031

**Table 3:** ESBL status among MDR and XDR *E. coli* isolates.

ESBL						
Resistance	Positive		Negative		Total	*p* value
	*N*	%	*N*	%		
MDR	48	96	16	69.5	64	0.001
XDR	2	4	7	30.4	9	

**Table 4:** Molecular prevalence of *bla*_CTX-M_ and *bla*_TEM_ genes among ESBL and non-ESBL *E. coli* isolates.

Drug-resistantenzyme	ESBLs genes					
	CTX-M		TEM		CTX-M + TEM	
	*N*	%	*N*	%	*N*	%
ESBL producer	25	50	23	46	13	26
ESBL nonproducer	11	47.8	17	73.9	11	47.8

**Table 5:** Diagnostic evaluation of the phenotypical method on comparison with detection of *bla*_CTX-M_ genes associated with ESBL in cephalosporin-resistant *E. coli*.

Test characteristics (molecular method)	Phenotypic method combined disk diffusion method
TP (PCR = 36)	25
FP (PCR negative)	23
TN (PCR = 37)	17
FN (PCR positive but phenotypic negative)	11
Sensitivity	69.44%, 95% CI (51.89% to 83.65%)
Specificity	42.50%, 95% CI (27.04% to 59.11%)
PPV	52.08%, 95% CI (43.54% to 60.51%)
NPV	60.71%, 95% CI (45.64% to 73.99%)
Accuracy	55.26%, 95% CI (43.41% to 66.69%)

TP = True Positive, FP = False Positive, TN = True Negative, FN = False Negative, PPV = positive predictive value, NPV = negative predictive value.

## Data Availability

All the data generated in our study are within the manuscript. The additional raw data have been uploaded in Supplementary File 1 and Supplementary File 2.

## Abbreviations

ATCC: American Type Culture Collection
CLSI: Clinical and Laboratory Standards Institute
MHA: Mueller–Hinton agar
CDM: Combined disk method
*bla*: *β*-lactamase coding gene
CTX-M: Cefotaximase-Munich
CFU: Colony-forming unit
ESBLs: Extended-spectrum beta-lactamases
GI: Gastro-intestinal
LF: Lactose fermenting
LPS: Lipopolysaccharide
MDR: Multidrug-resistant
XDR: Extensively drug-resistance
PDR: Pandrug-resistant
NLF: Nonlactose fermenting
PBP: Penicillin-binding proteins
PCR: Polymerase chain reaction.

## Supplementary Materials

Supplementary Materials The first file uploaded (Supplementary File 1) was the raw data of patient's age and sex and their antimicrobial susceptibility pattern of uropathogenic *E. coli*. The second file uploaded (Supplementary File 2) includes the raw data of the drug-resistant profile (MDR/XDR), phenotypical ESBL results, and the observed two ESBL genes (*bla*_CTX-M_ and *bla*_TEM_) of *E. coli*.
