# Supplementary material for: Prevalence of blaCTX-M and blaTEM Genes in Cefotaxime-Resistant Escherichia coli Recovered from Tertiary Care at Central Nepal: A Descriptive Cross-Sectional Study
Source: Can J Infect Dis Med Microbiol. 2024 Jan 8;2024:5517662. doi: 10.1155/2024/5517662 (PMC10789516; doi:10.1155/2024/5517662)
Supplement: Supplementary Materials — The first file uploaded (Supplementary File 1) was the raw data of patient's age and sex and their antimicrobial susceptibility pattern of uropathogenic E. coli. The second file uploaded (Supplementary File 2) includes the raw data of the drug-resistant profile (MDR/XDR), phenotypical ESBL results, and the observed two ESBL genes (blaCTX-M and blaTEM) of E. coli. [file 5517662.f1.zip › Supplementary file 1 (2).pdf]

Sample Code	Age/Sex	Antibiotics Used							
		Contrimoxazole	Gentamicin	Nitrofurantion	Norfloxacin	Meropenem	Ampicillin	Cefotaxime	Ceftazidime
1	33/F	Resistance	Sensitive	Sensitive	Resistance	Sensitive	Resistance	Resistance	Resistance
2	22/F	Sensitive	Sensitive	Sensitive	Sensitive	Sensitive	Resistance	Sensitive	Sensitive
3	16/F	Resistance	Sensitive	Sensitive	Resistance	Sensitive	Resistance	Sensitive	Sensitive
4	32/F	Resistance	Sensitive	Sensitive	Resistance	Resistance	Resistance	Resistance	Resistance
5	42/F	Sensitive	Sensitive	Sensitive	Resistance	Sensitive	Resistance	Resistance	Resistance
6	55/M	Resistance	Sensitive	Sensitive	Resistance	Sensitive	Sensitive	Resistance	Resistance
7	55/F	Sensitive	Sensitive	Sensitive	Sensitive	Sensitive	Resistance	Sensitive	Sensitive
8	28/F	Sensitive	Sensitive	Sensitive	Resistance	Sensitive	Resistance	Resistance	Resistance
9	32/M	Sensitive	Sensitive	Sensitive	Resistance	Sensitive	Sensitive	Resistance	Resistance
10	37/F	Resistance	Sensitive	Sensitive	Sensitive	Sensitive	Sensitive	Sensitive	Sensitive
11	29/F	Sensitive	Sensitive	Sensitive	Resistance	Sensitive	Resistance	Sensitive	Sensitive
12	17/F	Resistance	Sensitive	Sensitive	Sensitive	Sensitive	Resistance	Sensitive	Sensitive
13	57/F	Sensitive	Sensitive	Sensitive	Sensitive	Sensitive	Sensitive	Resistance	Resistance
14	18/F	Sensitive	Sensitive	Sensitive	Sensitive	Sensitive	Resistance	Sensitive	Sensitive
15	32/F	Resistance	Sensitive	Resistance	Resistance	Resistance	Resistance	Resistance	Resistance
16	47/F	Resistance	Resistance	Sensitive	Resistance	Sensitive	Resistance	Resistance	Resistance
17	78/F	Resistance	Sensitive	Sensitive	Sensitive	Sensitive	Resistance	Sensitive	Sensitive
18	44/F	Sensitive	Sensitive	Sensitive	Sensitive	Sensitive	Resistance	Sensitive	Sensitive
19	23/F	Resistance	Sensitive	Sensitive	Resistance	Sensitive	Resistance	Resistance	Resistance
20	25/F	Sensitive	Sensitive	Sensitive	Resistance	Sensitive	Sensitive	Sensitive	Sensitive
21	24/M	Sensitive	Sensitive	Sensitive	Sensitive	Sensitive	Sensitive	Sensitive	Sensitive
22	31/F	Sensitive	Sensitive	Resistance	Sensitive	Sensitive	Sensitive	Sensitive	Sensitive
23	68/M	Resistance	Sensitive	Sensitive	Resistance	Sensitive	Resistance	Resistance	Resistance
24	72/F	Resistance	Resistance	Sensitive	Resistance	Sensitive	Resistance	Resistance	Resistance
25	58/M	Resistance	Sensitive	Sensitive	Resistance	Sensitive	Resistance	Resistance	Resistance
26	58/F	Sensitive	Resistance	Sensitive	Sensitive	Sensitive	Sensitive	Sensitive	Sensitive
27	45/F	Resistance	Sensitive	Sensitive	Resistance	Sensitive	Resistance	Sensitive	Sensitive
28	55/F	Resistance	Resistance	Resistance	Resistance	Sensitive	Resistance	Resistance	Resistance
29	25/F	Sensitive	Sensitive	Sensitive	Sensitive	Sensitive	Resistance	sensitive	sensitive
30	39/F	Sensitive	Sensitive	Sensitive	Sensitive	Sensitive	Resistance	sensitive	sensitive
31	20/F	Sensitive	Sensitive	Sensitive	Sensitive	Sensitive	Sensitive	sensitive	sensitive

32	38/F	Sensitive	Sensitive	Sensitive	Sensitive	Sensitive	Sensitive	sensitive	sensitive
33	27/F	Sensitive	Sensitive	Sensitive	Sensitive	Sensitive	Resistance	Resistance	Resistance
34	88/F	Resistance	Sensitive	Sensitive	Resistance	Sensitive	Resistance	Resistance	Resistance
35	25/F	Sensitive	Sensitive	Sensitive	Sensitive	Sensitive	Resistance	Sensitive	Sensitive
36	67/F	Resistance	Sensitive	Resistance	Resistance	Sensitive	Resistance	Sensitive	Sensitive
37	73/F	Resistance	Sensitive	Sensitive	Resistance	Sensitive	Resistance	Sensitive	Sensitive
38	25/F	Sensitive	Sensitive	Sensitive	Sensitive	Sensitive	Resistance	Resistance	Resistance
39	62/M	Resistance	Sensitive	Resistance	Resistance	Resistance	Resistance	Resistance	Resistance
40	35/F	Resistance	Sensitive	Sensitive	Sensitive	Sensitive	Sensitive	Sensitive	Sensitive
41	21/F	Resistance	Sensitive	Sensitive	Sensitive	Sensitive	Sensitive	Sensitive	Sensitive
42	25/F	Resistance	Sensitive	Sensitive	Sensitive	Sensitive	Resistance	Sensitive	Sensitive
43	65/F	Resistance	Sensitive	Sensitive	Resistance	Sensitive	Resistance	Resistance	Resistance
44	63/F	Resistance	Sensitive	Sensitive	Resistance	Resistance	Resistance	Resistance	Resistance
45	24/F	Resistance	Sensitive	Sensitive	Sensitive	Sensitive	Sensitive	Sensitive	Sensitive
46	33/F	Resistance	Sensitive	Sensitive	Resistance	Sensitive	Resistance	Resistance	Resistance
47	59/F	Resistance	Resistance	Resistance	Resistance	Sensitive	Resistance	Resistance	Resistance
48	32/F	Sensitive	Sensitive	Sensitive	Sensitive	Sensitive	Sensitive	Sensitive	Sensitive
49	34/F	Sensitive	Sensitive	Sensitive	Sensitive	Sensitive	Sensitive	Sensitive	Sensitive
50	46/F	Sensitive	Sensitive	Sensitive	Sensitive	Sensitive	Sensitive	Sensitive	Sensitive
51	63/M	Resistance	Sensitive	Sensitive	Sensitive	Sensitive	Resistance	Resistance	Resistance
52	65/F	Resistance	Sensitive	Sensitive	Resistance	Sensitive	Resistance	Resistance	Resistance
53	75/F	Resistance	Resistance	Sensitive	Resistance	Resistance	Resistance	Resistance	Resistance
54	46/F	Resistance	Resistance	Sensitive	Resistance	Sensitive	Resistance	Resistance	Resistance
55	55/F	Resistance	Sensitive	Sensitive	Resistance	Sensitive	Resistance	Resistance	Resistance
56	36/F	Sensitive	Sensitive	Sensitive	Sensitive	Sensitive	Resistance	Resistance	Resistance
57	24/F	Resistance	Sensitive	Sensitive	Sensitive	Sensitive	Resistance	Sensitive	Sensitive
58	67/F	Sensitive	Sensitive	Sensitive	Sensitive	Sensitive	Sensitive	Sensitive	Sensitive
59	40/F	Sensitive	Sensitive	Sensitive	Resistance	Sensitive	Sensitive	Sensitive	Sensitive
60	44/M	Resistance	Sensitive	Sensitive	Resistance	Sensitive	Resistance	Sensitive	Sensitive
61	81/M	Resistance	Sensitive	Sensitive	Resistance	Sensitive	Resistance	Resistance	Resistance
62	25/F	Resistance	Sensitive	Sensitive	Sensitive	Sensitive	Sensitive	Sensitive	Sensitive
63	34/F	Resistance	Sensitive	Sensitive	Resistance	Sensitive	Resistance	Resistance	Resistance
64	42/F	Resistance	Sensitive	Sensitive	Resistance	Sensitive	Resistance	Resistance	Resistance
65	72/F	Sensitive	Sensitive	Resistance	Sensitive	Sensitive	Sensitive	Sensitive	Sensitive

66	34/F	Sensitive	Sensitive	Sensitive	Sensitive	Sensitive	Sensitive	Sensitive	Sensitive
67	30/F	Sensitive	Sensitive	Sensitive	Sensitive	Sensitive	Sensitive	Sensitive	Sensitive
68	23/F	Resistance	Sensitive	Resistance	Resistance	Sensitive	Sensitive	Sensitive	Sensitive
69	40/F	Resistance	Sensitive	Resistance	Sensitive	Sensitive	Resistance	Resistance	Resistance
70	7/F	Resistance	Sensitive	Sensitive	Sensitive	Sensitive	Resistance	Resistance	Resistance
71	47/F	Resistance	Sensitive	Sensitive	Sensitive	Sensitive	Resistance	Sensitive	Sensitive
72	63/F	Resistance	Sensitive	Sensitive	Sensitive	Sensitive	Resistance	Sensitive	Sensitive
73	74/F	Resistance	Sensitive	Resistance	Resistance	Sensitive	Resistance	Resistance	Resistance
74	31/F	Sensitive	Sensitive	Sensitive	Resistance	Sensitive	Resistance	Sensitive	Sensitive
75	62/M	Resistance	Sensitive	Resistance	Resistance	Resistance	Resistance	Resistance	Resistance
76	76/F	Resistance	Resistance	Sensitive	Resistance	Sensitive	Resistance	Resistance	Resistance
77	43/M	Sensitive	Sensitive	Sensitive	Sensitive	Sensitive	Sensitive	Sensitive	Sensitive
78	50/F	Sensitive	Sensitive	Sensitive	Sensitive	Sensitive	Sensitive	sensitive	sensitive
79	57/F	Sensitive	Sensitive	Sensitive	Resistance	Sensitive	Resistance	Resistance	Resistance
80	47/F	Resistance	Sensitive	Resistance	Resistance	Sensitive	Sensitive	sensitive	sensitive
81	39/F	Resistance	Sensitive	Sensitive	Sensitive	Sensitive	Resistance	sensitive	sensitive
82	59/M	Resistance	Resistance	Sensitive	Resistance	Sensitive	Resistance	Resistance	Resistance
83	60/F	Resistance	Resistance	Sensitive	Resistance	Sensitive	Resistance	Resistance	Resistance
84	62/F	Resistance	Sensitive	Sensitive	Resistance	Sensitive	Resistance	Sensitive	Sensitive
85	63/F	Sensitive	Sensitive	Sensitive	Resistance	Sensitive	Resistance	Resistance	Resistance
86	74/F	Resistance	Sensitive	Sensitive	Sensitive	Sensitive	Resistance	Resistance	Resistance
87	48/F	Sensitive	Sensitive	Sensitive	Resistance	Sensitive	Resistance	Resistance	Resistance
88	44/F	Resistance	Sensitive	Sensitive	Resistance	Sensitive	Resistance	Resistance	Resistance
89	71/F	Sensitive	Sensitive	Sensitive	Sensitive	Sensitive	Sensitive	Sensitive	Sensitive
90	29/M	Resistance	Sensitive	Sensitive	Resistance	Sensitive	Resistance	Resistance	Resistance
91	42/F	Resistance	Sensitive	Sensitive	Sensitive	Sensitive	Sensitive	Sensitive	Sensitive
92	45/F	Resistance	Sensitive	Resistance	Sensitive	Sensitive	Resistance	Sensitive	Sensitive
93	61/F	Sensitive	Sensitive	Sensitive	Sensitive	Sensitive	Resistance	Resistance	Resistance
94	30/F	Resistance	Sensitive	Sensitive	Sensitive	Sensitive	Resistance	Resistance	Resistance
95	62/F	Resistance	Sensitive	Sensitive	Resistance	Sensitive	Resistance	Resistance	Resistance
96	27/F	Resistance	Sensitive	Sensitive	Sensitive	Sensitive	Resistance	Sensitive	Sensitive
97	70/F	Resistance	Sensitive	Sensitive	Resistance	Sensitive	Resistance	Resistance	Resistance
98	47/M	Sensitive	Sensitive	Sensitive	Sensitive	Sensitive	Resistance	Sensitive	Sensitive
99	40/F	Sensitive	Sensitive	Sensitive	Sensitive	Sensitive	Resistance	Sensitive	Sensitive

100	71/M	Sensitive	Sensitive	Sensitive	Sensitive	Sensitive	Sensitive	Sensitive	Sensitive
101	72/F	Resistance	Sensitive	Resistance	Resistance	Sensitive	Resistance	Sensitive	Sensitive
102	62/F	Resistance	Resistance	Sensitive	Resistance	Sensitive	Resistance	Resistance	Resistance
103	78/F	Resistance	Sensitive	Sensitive	Resistance	Sensitive	Sensitive	Sensitive	Sensitive
104	60/M	Resistance	Resistance	Sensitive	Resistance	Resistance	Resistance	Resistance	Resistance
105	64/F	Sensitive	Sensitive	Sensitive	Resistance	Sensitive	Resistance	Resistance	Resistance
106	87/F	Sensitive	Resistance	Resistance	Sensitive	Sensitive	Resistance	Resistance	Resistance
107	19/M	Sensitive	Sensitive	Resistance	Sensitive	Sensitive	Sensitive	Sensitive	Sensitive
108	68/M	Sensitive	Resistance	Sensitive	Resistance	Sensitive	Resistance	Resistance	Resistance
109	33/F	Resistance	Sensitive	Sensitive	Resistance	Sensitive	Resistance	Resistance	Resistance
110	80/F	Sensitive	Sensitive	Resistance	Resistance	Sensitive	Sensitive	Resistance	Resistance
111	30/F	Resistance	Sensitive	Resistance	Sensitive	Sensitive	Resistance	Sensitive	Sensitive
112	77/F	Resistance	Sensitive	Sensitive	Resistance	Sensitive	Sensitive	Resistance	Resistance
113	25/F	Sensitive	Sensitive	Resistance	Sensitive	Sensitive	Resistance	Sensitive	Sensitive
114	49/F	Resistance	Sensitive	Sensitive	Resistance	Sensitive	Sensitive	Resistance	Resistance
115	29/M	Resistance	Sensitive	Sensitive	Resistance	Sensitive	Resistance	Resistance	Resistance
116	54/F	Sensitive	Sensitive	Sensitive	Sensitive	Sensitive	Sensitive	Sensitive	Sensitive
117	25/F	Sensitive	Sensitive	Sensitive	Resistance	Sensitive	Sensitive	Resistance	Resistance
118	18/F	Sensitive	Sensitive	Sensitive	Resistance	Sensitive	Sensitive	Resistance	Resistance
119	45/F	Sensitive	Sensitive	Sensitive	Sensitive	Sensitive	Sensitive	Sensitive	Sensitive
120	63/F	Sensitive	Sensitive	Sensitive	Resistance	Sensitive	Sensitive	Resistance	Resistance
121	46/F	Sensitive	Sensitive	Sensitive	Sensitive	Sensitive	Sensitive	Sensitive	Sensitive
122	32/F	Sensitive	Sensitive	Sensitive	Sensitive	Sensitive	Sensitive	Sensitive	Sensitive
123	67/F	Sensitive	Sensitive	Sensitive	Sensitive	Sensitive	Resistance	Sensitive	Sensitive
124	52/M	Sensitive	Sensitive	Sensitive	Sensitive	Sensitive	Sensitive	Sensitive	Sensitive
125	75/F	Resistance	Resistance	Sensitive	Resistance	Resistance	Resistance	Resistance	Resistance
126	25/F	Sensitive	Sensitive	Sensitive	Sensitive	Sensitive	Resistance	Sensitive	Sensitive
127	74/F	Sensitive	Sensitive	Sensitive	Sensitive	Sensitive	Sensitive	Sensitive	Sensitive
128	2/F	Sensitive	Sensitive	Sensitive	Resistance	Sensitive	Sensitive	Sensitive	Sensitive
129	41/F	Sensitive	Sensitive	Sensitive	Sensitive	Sensitive	Sensitive	Sensitive	Sensitive
130	48/F	Sensitive	Sensitive	Sensitive	Sensitive	Sensitive	Sensitive	Sensitive	Sensitive
131	57/F	Sensitive	Sensitive	Sensitive	Sensitive	Sensitive	Sensitive	Sensitive	Sensitive
132	50/M	Sensitive	Sensitive	Sensitive	Sensitive	Sensitive	Sensitive	Sensitive	Sensitive
133	55/F	Sensitive	Sensitive	Sensitive	Sensitive	Sensitive	Sensitive	Sensitive	Sensitive

134	88/M	Resistance	Sensitive	Sensitive	Sensitive	Sensitive	Sensitive	Sensitive	Sensitive
135	20/F	Resistance	Sensitive	Sensitive	Sensitive	Sensitive	Sensitive	Sensitive	Sensitive
136	52/M	Resistance	Sensitive	Sensitive	Sensitive	Sensitive	Sensitive	Sensitive	Sensitive
137	42/F	Resistance	Sensitive	Resistance	Resistance	Sensitive	Sensitive	Sensitive	Sensitive
138	57/F	Resistance	Sensitive	Sensitive	Sensitive	Sensitive	Sensitive	Sensitive	Sensitive
139	62/M	Sensitive	Sensitive	Resistance	Resistance	Sensitive	Resistance	Resistance	Resistance
140	59/F	Resistance	Sensitive	Sensitive	Resistance	Sensitive	Resistance	Resistance	Resistance
141	46/M	Sensitive	Sensitive	Sensitive	Sensitive	Sensitive	Sensitive	Sensitive	Sensitive
142	90/F	Sensitive	Sensitive	Sensitive	Sensitive	Sensitive	Sensitive	Sensitive	Sensitive
143	66/M	Resistance	Resistance	Resistance	Resistance	Sensitive	Resistance	Sensitive	Sensitive
144	70/F	Sensitive	Sensitive	Sensitive	Sensitive	Sensitive	Sensitive	Sensitive	Sensitive
145	48/F	Resistance	Sensitive	Sensitive	Resistance	Sensitive	Sensitive	Sensitive	Sensitive
146	65/M	Sensitive	Sensitive	Sensitive	Sensitive	Sensitive	Sensitive	Sensitive	Sensitive
147	36/F	Sensitive	Sensitive	Sensitive	Resistance	Sensitive	Sensitive	Resistance	Resistance
148	67/F	Resistance	Resistance	Sensitive	Resistance	Sensitive	Resistance	Resistance	Resistance
149	30/F	Sensitive	Sensitive	Sensitive	Resistance	Sensitive	Resistance	Sensitive	Sensitive
150	66/F	Sensitive	Sensitive	Sensitive	Resistance	Sensitive	Sensitive	Resistance	Resistance
151	1/F	Sensitive	Sensitive	Sensitive	Sensitive	Sensitive	Sensitive	Resistance	Resistance
152	75/M	Resistance	Resistance	Sensitive	Resistance	Sensitive	Resistance	Resistance	Resistance
153	48/F	Resistance	Resistance	Resistance	Resistance	Sensitive	Resistance	Resistance	Resistance
154	52/M	Resistance	Sensitive	Sensitive	Sensitive	Sensitive	Resistance	Sensitive	Sensitive
155	1/M	Resistance	Sensitive	Sensitive	Sensitive	Sensitive	Sensitive	Sensitive	Sensitive
156	44/M	Resistance	Resistance	Sensitive	Resistance	Sensitive	Resistance	Sensitive	Sensitive
157	75/F	Resistance	Resistance	Resistance	Resistance	Resistance	Resistance	Sensitive	Sensitive
158	61/M	Resistance	Resistance	Sensitive	Resistance	Sensitive	Resistance	Sensitive	Sensitive
159	35/F	Sensitive	Sensitive	Resistance	Sensitive	Sensitive	Sensitive	Resistance	Resistance
160	43/F	Sensitive	Sensitive	Sensitive	Sensitive	Sensitive	Sensitive	Sensitive	Sensitive
161	6/M	Sensitive	Sensitive	Sensitive	Resistance	Sensitive	Sensitive	Sensitive	Sensitive
162	43/F	Sensitive	Sensitive	Sensitive	Sensitive	Sensitive	Sensitive	Sensitive	Sensitive
163	23/F	Resistance	Sensitive	Sensitive	Sensitive	Sensitive	Sensitive	Resistance	Resistance
164	35/M	Resistance	Sensitive	Sensitive	Resistance	Sensitive	Sensitive	Resistance	Resistance
165	52/F	Sensitive	Sensitive	Sensitive	Sensitive	Sensitive	Sensitive	Sensitive	Sensitive

