# Supplementary material for: Prevalence of blaCTX-M and blaTEM Genes in Cefotaxime-Resistant Escherichia coli Recovered from Tertiary Care at Central Nepal: A Descriptive Cross-Sectional Study
Source: Can J Infect Dis Med Microbiol. 2024 Jan 8;2024:5517662. doi: 10.1155/2024/5517662 (PMC10789516; doi:10.1155/2024/5517662)
Supplement: Supplementary Materials — The first file uploaded (Supplementary File 1) was the raw data of patient's age and sex and their antimicrobial susceptibility pattern of uropathogenic E. coli. The second file uploaded (Supplementary File 2) includes the raw data of the drug-resistant profile (MDR/XDR), phenotypical ESBL results, and the observed two ESBL genes (blaCTX-M and blaTEM) of E. coli. [file 5517662.f1.zip › Supplementary file 2 (1).pdf]

Sample Code	Drug Resistance Pattern	Extended Spectrum Beta Lactamase	Extended Spectrum Beta Lactamase's Gene	
			CTX-M	TEM
1	Multi drug resistance	Detected	Detected	Detected
2	None	Not Applicable	Not Applicable	Not Applicable
3	Multi drug resistance	Not Applicable	Not Applicable	Not Applicable
4	Extensive drug resistance	Not detected	Detected	Detected
5	Multi drug resistance	Detected	Not detected	Not detected
6	Multi drug resistance	Detected	Detected	Detected
7	None	Not Applicable	Not Applicable	Not Applicable
8	Multi drug resistance	Detected	Detected	Not detected
9	Multi drug resistance	Detected	Detected	Detected
10	None	Not Applicable	Not Applicable	Not Applicable
11	None	Not Applicable	Not Applicable	Not Applicable
12	None	Not Applicable	Not Applicable	Not Applicable
13	Multi drug resistance	Detected	Detected	Detected
14	None	Not Applicable	Not Applicable	Not Applicable
15	Extensive drug resistance	Not detected	Detected	Detected
16	Multi drug resistance	Detected	Detected	Not detected
17	None	Not Applicable	Not Applicable	Not Applicable
18	None	Not Applicable	Not Applicable	Not Applicable
19	Multi drug resistance	Detected	Detected	Detected
20	None	Not Applicable	Not Applicable	Not Applicable
21	None	Not Applicable	Not Applicable	Not Applicable
22	None	Not Applicable	Not Applicable	Not Applicable
23	Multi drug resistance	Detected	Detected	Detected
24	Multi drug resistance	Detected	Not detected	Not detected
25	Multi drug resistance	Detected	Detected	Detected
26	None	Not Applicable	Not Applicable	Not Applicable
27	Multi drug resistance	Not Applicable	Not Applicable	Not Applicable
28	Multi drug resistance	Detected	Not detected	Detected
29	Multi drug resistance	Not Applicable	Not Applicable	Not Applicable
30	None	Not Applicable	Not Applicable	Not Applicable
31	None	Not Applicable	Not Applicable	Not Applicable

32	None	Not Applicable	Not Applicable	Not Applicable
33	None	Detected	Not detected	Detected
34	Multi drug resistance	Detected	Detected	Detected
35	None	Not Applicable	Not Applicable	Not Applicable
36	Multi drug resistance	Not Applicable	Not Applicable	Not Applicable
37	Multi drug resistance	Not Applicable	Not Applicable	Not Applicable
38	Multi drug resistance	Detected	Detected	Not detected
39	Extensive drug resistance	Not detected	Not detected	Detected
40	None	Not Applicable	Not Applicable	Not Applicable
41	None	Not Applicable	Not Applicable	Not Applicable
42	None	Not Applicable	Not Applicable	Not Applicable
43	Multi drug resistance	Detected	Detected	Not detected
44	Multi drug resistance	Not detected	Not detected	Not detected
45	None	Not Applicable	Not Applicable	Not Applicable
46	Multi drug resistance	Detected	Detected	Not detected
47	Multi drug resistance	Not detected	Not detected	Detected
48	None	Not Applicable	Not Applicable	Not Applicable
49	None	Not Applicable	Not Applicable	Not Applicable
50	None	Not Applicable	Not Applicable	Not Applicable
51	Extensive drug resistance	Detected	Not detected	Not detected
52	Multi drug resistance	Detected	Detected	Not detected
53	Extensive drug resistance	Not detected	Not detected	Not detected
54	Multi drug resistance	Detected	Not detected	Not detected
55	Multi drug resistance	Detected	Not detected	Not detected
56	Multi drug resistance	Detected	Detected	Not detected
57	None	Not Applicable	Not Applicable	Not Applicable
58	None	Not Applicable	Not Applicable	Not Applicable
59	None	Not Applicable	Not Applicable	Not Applicable
60	Multi drug resistance	Not Applicable	Not Applicable	Not Applicable
61	Multi drug resistance	Detected	Detected	Detected
62	None	Not Applicable	Not Applicable	Not Applicable
63	Multi drug resistance	Not detected	Not detected	Not detected
64	Multi drug resistance	Detected	Detected	Not detected
65	None	Not Applicable	Not Applicable	Not Applicable

66	None	Not Applicable	Not Applicable	Not Applicable
67	None	Not Applicable	Not Applicable	Not Applicable
68	Multi drug resistance	Not Applicable	Not Applicable	Not Applicable
69	Multi drug resistance	Detected	Not detected	Not detected
70	Multi drug resistance	Not detected	Not detected	Detected
71	None	Not Applicable	Not Applicable	Not Applicable
72	None	Not Applicable	Not Applicable	Not Applicable
73	Multi drug resistance	Not detected	Not detected	Not detected
74	Multi drug resistance	Not Applicable	Not Applicable	Not Applicable
75	Extensive drug resistance	Not detected	Detected	Detected
76	Multi drug resistance	Detected	Not detected	Not detected
77	Multi drug resistance	Not Applicable	Not Applicable	Not Applicable
78	None	Not Applicable	Not Applicable	Not Applicable
79	Multi drug resistance	Detected	Detected	Not detected
80	Multi drug resistance	Not Applicable	Not Applicable	Not Applicable
81	Multi drug resistance	Not Applicable	Not Applicable	Not Applicable
82	Extensive drug resistance	Detected	Not detected	Not detected
83	Multi drug resistance	Detected	Not detected	Detected
84	Multi drug resistance	Not Applicable	Not Applicable	Not Applicable
85	Multi drug resistance	Detected	Not detected	Not detected
86	Multi drug resistance	Detected	Detected	Not detected
87	Multi drug resistance	Detected	Detected	Not detected
88	Multi drug resistance	Detected	Not detected	Detected
89	None	Not Applicable	Not Applicable	Not Applicable
90	Multi drug resistance	Not detected	Detected	Detected
91	None	Not Applicable	Not Applicable	Not Applicable
92	Multi drug resistance	Not Applicable	Not Applicable	Not Applicable
93	Multi drug resistance	Detected	Not detected	Detected
94	Multi drug resistance	Detected	Not detected	Not detected
95	Multi drug resistance	Detected	Not detected	Detected
96	Multi drug resistance	Not Applicable	Not Applicable	Not Applicable
97	Multi drug resistance	Detected	Detected	Detected
98	None	Not Applicable	Not Applicable	Not Applicable
99	None	Not Applicable	Not Applicable	Not Applicable

100	None	Not Applicable	Not Applicable	Not Applicable
101	Multi drug resistance	Not Applicable	Not Applicable	Not Applicable
102	Multi drug resistance	Not detected	Detected	Detected
103	None	Not Applicable	Not Applicable	Not Applicable
104	Multi drug resistance	Not detected	Not detected	Detected
105	Multi drug resistance	Detected	Not detected	Not detected
106	Multi drug resistance	Detected	Not detected	Not detected
107	None	Not Applicable	Not Applicable	Not Applicable
108	Multi drug resistance	Detected	Detected	Not detected
109	Multi drug resistance	Detected	Not detected	Detected
110	Multi drug resistance	Not detected	Detected	Detected
111	Multi drug resistance	Not Applicable	Not Applicable	Not Applicable
112	Multi drug resistance	Detected	Not detected	Detected
113	Multi drug resistance	Not Applicable	Not Applicable	Not Applicable
114	Multi drug resistance	Not detected	Detected	Detected
115	Multi drug resistance	Not detected	Detected	Detected
116	None	Not Applicable	Not Applicable	Not Applicable
117	Multi drug resistance	Not detected	Not detected	Not detected
118	Multi drug resistance	Detected	Not detected	Not detected
119	None	Not Applicable	Not Applicable	Not Applicable
120	Multi drug resistance	Not detected	Not detected	Not detected
121	None	Not Applicable	Not Applicable	Not Applicable
122	None	Not Applicable	Not Applicable	Not Applicable
123	None	Not Applicable	Not Applicable	Not Applicable
124	None	Not Applicable	Not Applicable	Not Applicable
125	Extensive drug resistance	Not detected	Not detected	Detected
126	None	Not Applicable	Not Applicable	Not Applicable
127	None	Not Applicable	Not Applicable	Not Applicable
128	None	Not Applicable	Not Applicable	Not Applicable
129	None	Not Applicable	Not Applicable	Not Applicable
130	None	Not Applicable	Not Applicable	Not Applicable
131	None	Not Applicable	Not Applicable	Not Applicable
132	None	Not Applicable	Not Applicable	Not Applicable
133	None	Not Applicable	Not Applicable	Not Applicable

134	None	Not Applicable	Not Applicable	Not Applicable
135	None	Not Applicable	Not Applicable	Not Applicable
136	None	Not Applicable	Not Applicable	Not Applicable
137	Multi drug resistance	Not Applicable	Not Applicable	Not Applicable
138	None	Not Applicable	Not Applicable	Not Applicable
139	Multi drug resistance	Detected	Detected	Detected
140	Multi drug resistance	Not detected	Not detected	Detected
141	None	Not Applicable	Not Applicable	Not Applicable
142	None	Not Applicable	Not Applicable	Not Applicable
143	Extensive drug resistance	Not Applicable	Not Applicable	Not Applicable
144	None	Not Applicable	Not Applicable	Not Applicable
145	None	Not Applicable	Not Applicable	Not Applicable
146	None	Not Applicable	Not Applicable	Not Applicable
147	Multi drug resistance	Not detected	Detected	Detected
148	Multi drug resistance	Detected	Not detected	Not detected
149	Multi drug resistance	Not Applicable	Not Applicable	Not Applicable
150	Multi drug resistance	Detected	Detected	Detected
151	Multi drug resistance	Detected	Not detected	Not detected
152	Multi drug resistance	Detected	Not detected	Detected
153	None	Not Applicable	Not Applicable	Not Applicable
154	Multi drug resistance	Not Applicable	Not Applicable	Not Applicable
155	None	Not Applicable	Not Applicable	Not Applicable
156	Multi drug resistance	Not Applicable	Not Applicable	Not Applicable
157	Extensive drug resistance	Not detected	Detected	Detected
158	Multi drug resistance	Not detected	Not detected	Not detected
159	Multi drug resistance	Detected	Not detected	Detected
160	None	Not detected	Not detected	Not detected
161	None	Not detected	Not detected	Not detected
162	None	Not detected	Not detected	Not detected
163	Multi drug resistance	Detected	Detected	Detected
164	Multi drug resistance	Not detected	Detected	Detected
165	None	Not detected	Not detected	Not detected
